# Periscope; or, Circumspect Review

**Published:** 1828-04-01

**Authors:** 


					, l8283 ( 48i )
OR,
CIRCUMSPECTIVE REVIEW.
Ore trahit quodcunque potest, atque addit acervo."
[Oth FEBRUARY, 1828.]
1. Thoracic Diseases.
In a late sitting of the Medico-Chirurgi-
cal Society (22nd. Jan.) the President,
Mr. Travers, communicated two cases of
Medullary disease in the chest, which led
to some interesting observations from
three or four of the members present.
It is not our intention to give any details
?f the papers read at this Society, seeing
that they will now be regularly published
every year; but in the present instance
We cannot notice the oral discussions,
Without some particulars of the cases that
gave origin to them.
The first case was one that occurred at
St. George's Hospital, about a year ago,
and of which we took some notice at the
time. A man came in under Dr. Hevvett,
With evident symptoms of watery effu-
sion in the left side of the chest, in which
Ro respiratory sound could be heard. The
heart was found beating in the right
side, and the sound, on percussion, was
dull on that side. The poor man being
threatened with suffocation, Mr. Brodie
performed paracentesis thoracis, and
drew off a very great quantity of sero-
sanguineous fluid. A temporary mitiga-
tion of the symptoms ensued ; but, in two
?r three days, the patient died. Mean-
time, the heart returned to its natural
position, when the fluid was drawn off.
t)n dissection, a fungoid tumour, of ma-
lignant character, was found growing
from the diaphragm, and projecting into
the left side of the thorax. The lungs
Were congested, but there was 110 other
Material disease.
The second case was one in private
practice. A young gentleman, till with-
in three weeks of his death, was sup-
posed to'be in perfect health, and could
tire out men and horses in the sports of
the field. About Christmas, 1827, a
sudden stop was put to these exercises,
by pain in the right side, difficulty of
breathing, and other phenomena which
induced the medical attendants to con-
sider the liver in fault. In a short time
afterwards there were pretty strong evi-
dences of a fluid effused in the right
pleural cavity, evinced by a bulging out
of that side?by an inability to lie on the
opposite side?and, in short, by distinct
fluctuation in that side. A trocar was
introduced, and a large quantity of fluid,
of a clear straw-colour, was drawn off.
Great relief was thus obtained?and con-
siderable hopes of cure ivere entertained
by the medical attendants, and communi-
cated to the friends. But these hopes
were soon dissipated. The chest filled
again, and the young gentleman breathed
his last, on the road to London, in about
three weeks from the apparent com-
mencement of the disease. On dissec-
tion, there was found a very large me-
dullary fungus occupying the whole of
the upper portion of the right lung, and
weighing about three pounds?effusion
into that side of the chest?universal tu-
berculation of the pleura costalis and
pulmonalis of that side?an almost com-
plete obliteration of the left lung, which
was reduced to a condensed lamina,
three lines in thickness, adherent to the
left pleura costalis. Thus, the only por-
tion of respifable lung, was the inferior
lobe on the right side. There was no
disease in any of the abdominal viscera.
No mention of auscultation or percussion
was made in the paper ; but the Presi-
dent requested that members would give
their opinions how far the stethoscope
might have elucidated the pathological
condition of the chest. Dr. Johnson ob-
served that, as only the inferior lobe of the
right lung was in a respirable state, so,
both auscultation and percussion would
have proved that fact, without the small-
est difficulty. It was quite impossible that
Vol. VIII. No 10. 3 II
482 Periscope; or, Circumspective Review. [Feb. 0
the respiratory murmur could hare been
heard in any part of the chest, except in
what was occupied by the said lobe. So
far auscultation and percussion might
have prevented the favourable prognosis
which was given to the friends. And now
a ruse de guerre was played off, and a
masked battery opened against ausculta-
tion. Mr. Travers immediately stated
that, " agentleman who carried a stethos-
cope, gave a most favourable prognosis
of the case." A smile of congratulation
mantled on the animated countenances
of the anti-auscultators?and, while Mr.
Travel's was pronouncing sentence on
the stethoscope, their ears could actually
be seen to stand up?we will not venture
to say how far beyond their usual longi-
tudes. But the ruse de guerre failed?
the masked battery missed fire, and the
trap which was laid for auscultation
was occupied with other subjects. Dr.
Johnson instantly put the question to the
President, whether, by this verbal state-
ment, (not on the record of the case,)
he meant to throw the error of prognosis
on auscultation, or on the auscultator?
on the measure, or on the man ? The
President candidly attributed the error
to the man ! The dimpling smiles faded
at once?and not a single anti-auscul-
tator even attempted to invalidate the
position maintained by Dr. Johnson.
Mr. Lawrence adverted to the ques-
tion, " was this immense quantum of
disease developed in the short space of
three weeks, or might the origin of it be
dated back to a former period, when
this young gentleman had had a small
medullary tumour removed from one of
the lower extremities ?" an event we
forgot to mention in the narrative. Mr.
L- was inclined to suspect that the dis-
ease did not exist prior to the recent
fatal illness?and he was led to this sus-
picion, by the rapid progress which me-
dullary diseases usually make. In illus-
tration of this, he related the case of a
young gentleman who complained of pain
in his knee, about three months before
his death, without any visible affection of
the part.* This increased?a swelling,
of a pulsatile kind, took place?and, in
three months, his health was so complete-
ly broken up, that amputation was per-
formed to give him a hope of preservation.
The operation did not save him. On dis-
section, a medullary tumour was found to
have commenced between the heads of
the tibia and fibula, involving all the
contiguous parts in destruction. Mr.
L. related another case, which had very
little reference, except to Dr. Hewett's
case, where the heart was displaced. A
boy had been jammed between a cart and
a post, and was brought into Bartholo-
mew's, in a state of great depression,
from which he was not expected to re-
cover. In a few days, however, a smart
re-action came on, and now it was dis-
covered that the heart was beating tre-
mendously in the right side of the thorax.
Mr. L. was inclined, at first, to look on
this as a malformation, or transposition.
But, in process of time, the heart gradu-
ally came round to its proper place in
the left side, and the boy recovered. On
being asked by Mr. Earle, if the stethos-
cope had been applied in this case, Mr. L.
answered no :?the phenomena were so
evident to the senses, that auscultation
and percussion were unnecessary. Mr.
L. was then asked by Dr. Johnson,
" what it was that had displaced the
heart, seeing that the phenomena were
all so evident ?" Mr. L. replied that he
could not tell. So then, Mr. L. knew
that the heart was in the right (or rather
in the wrong) side ? but what was in
the left side of the chest God only knew.
It might be air from a fractured rib and
wounded lung?or it might be a serous,
or sanguineous effusion, but as the nature
of the case was so evident to the senses,
the left side of the chest was never exa-
mined by auscultation or percussion ! !
Mr. Earle, in illustration of the rapid
growth of these medullary tumours, re-
lated the case of a young gentleman,
from whom he removed a small tumour
of this kind, a few months ago, which
was seated over the parotid gland. He
was then in apparent good health, but
has since died, presenting a great num-
ber of tumours in the liver and abdomen
generally. The body, however, had not
been examined. Notwithstanding these
proofs of the rapidity of growth, in medul-
lary tumours, we cannot agree with Mr.
Lawrence in believing that, in the se-
* We were informed by a young gen-
tleman in the Society, who knew the pa-
tient, that the latter had complained for
a much longer time than three months,
of the pain in his knee.
1828] Foreign Bodies in Bodies Natural. 483
cond case, the mischief might be pro-
duced in three weeks. The immense
size of the principal tumour?the com-
plete tuberculation of the pleura?the
annihilation of the left lung?and the
serous effusion, were the work of longer
time.
There was one other subject mooted
at a late hour in the Society, which we
deem worthy of notice, on account of
the confident, but, we apprehend, some-
what hasty opinion, delivered by Mr.
Lawrence. The President asked the
Society whether it was consonant with
their experience, to find dropsical effu-
sion in the chest, independent of organic
disease. Mr. Lawrence argued that
such effusion did not exist, independent
?f organic affection. This opinion we
hold to be erroneous. There are several
states of the system, especially inflam-
matory states, where serous effusion takes
place into the pleural as well as into other
cavities of the body, totally independent
?f organic disease, in any of the vital
prgans. Effusion obtains, more or less,
in all severe attacks of pleuritis?which,
conclude, Mr. Lawrence would not
designate as an organic disease. The
same takes place in peritonitis, into the
abdomen?and into the ventricles of the
hrain, in meningeal inflammation. But
there is the very best reason to believe
that, without some inflammation, there
luay be a dropsical effusion into the chest,
and other parts of the body?as, for ex-
ample, after severe haemorrhages, from
Occidents or operations. These consi-
derations induce us to dissent from the
?pinion delivered by Mr. Lawrence?par-
ticularly as it is a doctrine which leads
t? despondency in the treatment, and
takes away all hope from a surgical oper-
ation.
2. Foreign Bodies in Bodies
Natural.*
Some curious cases have lately been given
to the public, by our esteemed contempo-
rary, where pins, needles, teeth of combs,
have been taken out of various parts
the body, no account being furnished
of how they got hi. The first was that
of a girl, admitted into the Middlesex
Hospital, with violent pain in the belly.
On examination, there was found, a little
below the umbilicus, a hard point, arid
on making an incision, out started the
head of a pin! It was taken hold of by
the forceps, and drawn out one?two?
three?five inches?'and, at last, on com-
ing away, proved to be a brass hat-pin,
six inches in length ! The young woman,
of course, had not the remotest idea how
it could possibly have come there, unless,
indeed, she had swallowed it by mistake.
This case is followed by another, related
by Mr. Brodie himself. Mrs. H. " a
middle-aged woman," (was she married?)
was subject to hysteria and pain in the
side. One evening she called up her
maid servant, and told her that she had
dropped a paper, containing fifty needles,
upon the foot stool, and thought that
some of them had stuck into her leg.
Her medical attendant was called, and
extracted two small ones, from a little
above the ankle; but eight only of the
fifty could be found in the paper ! In a
few days, Mr. Brodie was sent for, ard
removed, at intervals, twenty-six needles
from the legs, which became cedematous,
with much general disturbance of the
health. One evening, towards the end
of June, she fell into a state of insensi-
bility, and at two o'clock next morning
expired. On examination, the leg from
which the needles had been taken was
found loaded with lymph and serum, and
sufficient needles were found still im-
bedded in the cellular tissue to account
for nearly the whole number missing.
Mr. Brodie hazards no conjecture upon
the mode in which this paper of needles
was disposed of. That he imagines they
actually did " stick into" the foot, and
travel thence up the leg, we cannot be-
lieve, but we suppose that such being the
account given, Mr. B's well-known gal-
lantry would prevent his doubting the
lady's word, whatever opinion he might
hold to the contrary.*
Mr. Brodie is followed by Mr. Bell, who
somewhat startled us by stating, that" the
* London Med. Gazette, Nos. 3, 6, 7j
passim.
* No two persons agree in their inter-
pretation of this paper of Mr. Brodie's.
One party thinks that the lady swallowed
the pins, the other that they got into
the membe vify the foot. For our parts,
3 H 2
484 Periscope; or, Circumspective Review. [Feb. 9
letter of Mr. Brodie curiously explains,
"how needles may get into the body."
The deuce it does ! Now, to our minds,
it seems to be much more satisfactory as
to the methods of getting them out. But
to the point. A farmer's wife, accom-
panied by her crony, came to Mr. Bell,
complaining of excessive pain and prick-
ing in her hip, near the left labium.
After much to do, he was allowed to ex-
amine, when he found an abscess point-
ing, with something like a foreign body
in it. He opened the abscess, and ex-
tracted four of the coarse teeth of a dres-
sing comb. Mr. B. was surprised, but
the good ladies were absolutely petrified,
and, all at once, became exceeding anxious
to depart, assigning no reason whatever
for the presence of the body in that situ-
ation. Mr. Bell observes, that bones oc-
casionally lodge in the rectum, and excite
great irritation in the bladder. One case
was mistaken for cancer, but, on ex-
tracting the bone, all the symptoms dis-
appeared. In another instance, a phleg-
monous tumour formed on the hip, which
was opened, and a great many bones of
small birds taken away. Here, however,
there was scirrhous contraction besides.
Whenever, during stricture of the rec-
tum, there is much increase of pain, &c.
Mr. Bell recommends introducing a sound,
to detect any substances which may have
been entangled abov? the obstruction.
We might relate s veral other instances
of the introduction v,f foreign bodies into
the " human form divine," but it would
be loading our columns, and wearying
the patience of our readers, were we to
chronicle all the strange antics which
hysteric females play.
WESTMINSTER MEDICAL SOCIETY.
3. Delirium Tremens.
On Saturday night (26th January) an
interesting discussion took place on this
dangerous disease. The subject was in-
troduced by Mr. Hunt, who gave a very
concise account of five cases, three of
which, we understood, proved fatal.
These were treated chiefly on the anti-
phlogistic plan. In these cases, there
were febrile symptoms, and some other
phenomena, which threw a suspicion on
the fact of their being pure specimens of
the disease. The fifth case was more
particularly detailed, and the patient had
several attacks of this peculiar malady,
from which he recovered by small de-
tractions of blood, in the first instance,
and then opium and other diffusible sti-
muli. This was the mode of treatment
which the author recommended, steering
a middle course between those who look
upon the disease as an inflammatory
affection of the brain or its membranes,
and those who consider the phenomena as
dependent on an exhausted condition of
the sensorial energy. In one dissection
made by Mr. Hunt, there were marks of
inflammation in the brain, as coagulable
lymph and serous effusion.
The discussion then turned on the
pathology of the disease. Dr. Johnson
maintained, from many cases which he
had seen of delirium tremens, and some
dissections, that pure specimens of the
disease were not necessarily connected
with inflammation of the brain, and, con-
sequently, that the basis of the treatment
was opium to procure sleep, and diffusible
stimuli to equallize the circulation and
excitement. He stated two cases that
had recently fallen under his observation,
and in one of these, the most minute
dissection could not detect the slightest
mark of turgescence, or previous in-
creased action in the brain or its mem-
branes. A gentleman who resided in a
northern part of the kingdom, where the
disease is very common, stated that dif-
fusible stimuli, and more especially large
doses of ammonia, were found the only
remedies on which any reliance could be
placed. Mr. Lambert asked if a disease
resembling the one under consideration,
did not often occur, after wounds and
accidents ? This was admitted by Dr.
Johnson, Dr. Stewart and others. It
constituted the delirium traumaticum,
of Dupuytren, described in our first
Fasciculus. Dr. Stewart remarked that
he had seen the disease on a large
scale, in the United States of America,
and that almost all those patients who
were treated on the antiphlogistic plan,
died, while those who were treated
we think they made their entrance neither
by the stomach nor the toes, but, as in
most other instances of the kind, were
bona (or rather mala) fide introduced into
the part where they were found.
1828] Disease op the Pericranium. 485
by opium and stimulants, recovered.
Dr. Copland related some cases which he
had recently witnessed. They were saved
by moderate local depletion?calomel and
opium?and then smart doses of castor-
oil and oil of turpentine. In these cases,
there was much epigastric tenderness,
and the motions were very black and
fetid. The most important practical in-
formation, however, was elicited from
Dr. Ayre, who, while practising in Hull,
had extensive opportunities of witnessing
the complaint. He agreed entirely with
Dr. Johnson, as to the pathology of the
disease. He considered it the very re-
verse of inflammation. He observed it
to arise from several other causes than
intemperance in drink, though this last
?was the most frequent of all causes. He
had seen it result from the emanations of
lead?from starvation?and from some
other sedative causes. In the mode of
treatment, he differed somewhat from
those who had delivered their opinions in
the Society. The plan which he had
found most successful was that of giving
opium, not in very large doses, but in
smaller, and very frequent ones, com-
bining the opium with diffusible stimuli,
especially wine or spirits, whichever the
patient had been most accustomed to
before the commencement of the disease.
Dr. Ayre mentioned several curious cases
of this mysterious malady. He was asked
what were the pathognomonic features of
the disease. He answered, that the tre-
mor of the hands, the coolness of the
skin, the perspiration, the irascibility of
the temper, the loquacity of speech, and,
above all, the false images that were pre-
sented to the mind's eye of the patient,
Were the distinctive characters of delirium
tremens. There were several pertinent
remarks made by other members of the
Society, as Dr. Gregory, Dr. Sheil, Mr.
Lambert, Mr. Mackelcan, Mr. Chin-
nock, &c. but the general experience of
the Society was evidently in favour of the
stimulant and narcotic treatment, in un-
complicated cases of delirium tremens.
The pathology of the disease was uni-
versally pronounced to be that which was
stated by Drs. Johnson and Ayre.
Mr. Duncan begged to trespass one
moment on the Society, in order to in-
quire what were the appearances on dis-
section of. Nowlands, the young man,
whose carotid arteiy Mr. Wardrop had
tied, for an aneurismal tumour on the
head. The facts which we stated on the
cover of our last Fasciculus were fully
verified by Dr. Somerville, the President,
and Mr. Arnott, who, as well as Dr. John-
son, had inspected the parts.
4. Disease of the Pericranium.
The affection to which Dr. Abercrombie
has lately alluded, in his work on Diseases
of the Brain and Spinal Marrow, appears
to correspond with the periostitis of Dr.
Crampton, as described in the 1st volume
of the Dublin Hospital Reports. Sir
Everard Home published a paper, many
years ago, in which several cases of this
disease are detailed. The symptoms, in
these cases, were head-ach, with various
uneasy feelings about the head?painful
tenderness of the scalp in some particular
part, with some degree of swelling or
thickening of the integuments there. In
several cases, there were epileptiform fits.
They were treated by dividing the integu-
ments and pericranium freely down to
the bone, and then allowing the wound
to heal slowly. In making these inci-
sions, the pericranium was found mor-
bidly sensible, as well as thickened?and,
in some cases, indurated, so as to resemble
cartilage. This treatment by incisions
was followed, in some instances, by im-
mediate relief?in others, the patient re-
mained liable to fits or head-achs after
any excess or irregularity. In some of
them, the incision healed without any
affection of the bone being discovered?
in others, a portion of the bone appeared
white and porous,or honey-combed, and a
limpid fluid appeared to percolate through
it. In one of these cases, the porous
piece of bone exfoliated after the wound
had been dressed with dry lint for six
weeks. In one fatal case, Sir Everard
Home found the pericranium thickened
into a mass of a fibro-osseous texture,
and corresponding to this internally, there
was a similar thickening and induration
of the dura mater. Most of these cases
had been treated by long courses of mer-
cury, without success.
Mr. Crampton, among other cases, re-
lates one or two where the pericranium
was affected. A boy, aged 14 years, had
a small angry tumour on one side of the
486 Periscope; or, Circumspective Review. [Feb. 9
nose, which extended to the forehead,
with erysipelas and fever. On the 9th
day he became suddenly comatose, then
convulsed?and soon died. On dissec-
tion, the pericranium of the frontal bone
was found red, thickened, and detached
from the bone, there being much puru-
lent matter lying between them. The
dura mater was detached from a corres-
ponding space of bone internally, and a
greenish fluid was effused between them.
Some other cases are quoted by Dr. Aber-
crombie, who does not introduce any inr
stance as happening in his own practice.
5. Coup de Soleil, on Ictus Solaris.
In a long, but certainly not uninteresting
paper on this subject, in our Northern
cotemporary, Mr. Mitchell, a surgeon in
the navy, has given the results of his
experience in this curious complaint. It
was on the banks of Lake Champlain, in
Canada, that Mr. Mitchell's observations
were chiefly made, in the Summer of
1815. He had a great number of cases
of ictus solaris under his care?almost all
marines, who were taken ill on their
posts, with their muskets in their hands:
?" aud such was the fury with which
they were seized, that they actually made
a charge." He observed three kinds of
this disease?1st. Sudden and complete
insensibility, with total loss of power,
stupor, and stertorous breathing?the
apoplectic species s 2dly, with symptoms
of violent phrenitis and delirium : 3dly,
with symptoms of chronic phrenitis. It
is the first species that we shall notice,
as it is that which most commonly at-
tracts attention, and which is most cer-
tainly traced to the cause in question.
It most comrrionly occurs in plethoric
habits?in men exposed on service (with
the stomach distended with food, and the
vascular system excited by ardent spirits)
under a burning sun?or while sleeping
in a drunken fit, exposed to the solar
rays. In such cases, it is very difficult
to say whether apoplexy has actually taken
place, or whether the patient is merely
intoxicated. Our author does not attempt
to lay down any diagnostic marks. In
coup de soleil every thing depends on
?peedy and energetic treatment. Our
author effected a large depletion from
the jugular vein, in preference to any
other part?then shaved the head, and
poured on it cold fluids from a height?
thirdly, he placed the lower extremities
in very hot water?fourthly, administered
stimulating purgative enemata?fifthly,
he caused the whole body to be rubbed
with stimulating embrocations. If these
means prevent a fatal termination in the
onset, the after-treatment is the same as
for the other species. These other spe-
cies assimilate with acute and chronic
phrenitis, the treatment of which need
not be detailed.
A curious pathological investigation
would be the modus operandi of solar
heat in producing the ictus solaris. The
following passage expressive of the au-
thor's own feelings on an apparent ac-
cession of this affection, may not be with-
out interest.
" On one occasion, when having landed
on an island in Jarvis's Straits, which
was hardly any thing but a naked rock,
and that nearly white from the dung of
sea-birds, I suffered much from the re-
flection of a burning sun, and indeed I
thought I was on the point of having a
stroke of the sun, as I nearly lost my
vision, and my head felt distended as if
ready to burst. I rushed towards a cave,
in the rock facing the sea, and, finding
salt water in it, I took an handkerchief,
and with it kept my head in a constant
state of evaporation." In a quarter of
an hour he felt quite relieved.
The second species bears so much re-
semblance to acute phrenitis, that we
need not dwell upon it here. The treat-
ment must be very active. In one case
our author took away five pounds of blood
before deliquium animi could be induced,
and consequent reduction of arterial ex-
citement.
The third, or chronic species, was
found to come on gradually and insidi-
ously?and to continue a long time, with-
out destroying the powers of life. It had
a tendency " to disorder the arrangement
and association of ideas rather than to
destroy vitality." In all the cases he had
known, and where the patients had re-
turned to Europe, " their ideas never
became correct till they got out of the hot
into cooler latitudes, when they awoke,
as it were out of a dream, and wondered
how they had remained in such a delu-
sion." Mr. M. has only observed this
1828]- Encepiialoid Tumour in the Lungs. 487
species in hot climates. It evinced evi-
dent disorder or disease in the brain or
!ts membranes as well as in the digestive
organs. This species bears a consider-
able analogy to chronic forms of delirium
tremens. It is a temporary insanity,
from corporeal disease. The treatment
must be directed, of course, to the remo-
val of the physical disorder or disorders,
when the mental hallucinations will give
^vay. This paper is rather too much
spun out by Mr. Mitchell, though it is
indicative of good sense and careful ob-
servation.
G. ENCErHALOlD TUMOUH IN THE
Lungs.
We were much gratified to peruse a case
of this kind in our Gazette cotemporary,
from the pen of Dr. Seymour, though it
will deprive that talented physician of
the pleasure of sneering at auscultation,,
since it proves himself a cultivator of the
" inutile lignum." We suspect that a
great many will follow the example of
Dr. Seymour, and study auscultation and
percussion, instead of attempting?vainly
attempting, to " roll back the tide of
knowledge to its source," and check the
growth of improvements, so much needed,
in the healing art.
A man presented himself to Dr. Sey-
mour, with difficulty of breathing, violent
fits of coughing, scanty expectoration,
" wheezing noise on (in) the right side
of the chest," which rises and falls in
breathing, the ribs of the left side being
fixed?" and the left lung impervious to
airy* The pulse was 90 and weak, the
tongue clean and moist?no hectic fever.
" Complains of weight and tightness at
the scrobiculus cordis, and want of appe-
tite?bowels regular." An emetic was
exhibited, 15th November. Next day,
the sense of weight and tightness in epi-
gastrio was relieved?the expectoration
frothy?the respiration in the right side
" attended with a peculiar harsh noise,
like the blowing of bellows." " On the
left side the sound is perfectly dull." The
Doctor employs percussion?and much to
his credit. We cannot, however, see the
accordance of the emetic with the phy-
sical and other signs exhibited the pre-
ceding day. There was the most com-
plete evidence of extensive disease in the
chest?but none (to our humble appre-
hension) of a disordered stomach, the.
tongue being " clean and moist?the
bowels regular." If Dr. S. considers
weight and tightness at the scrobiculus.
cordis, with such evidence of thoracic
disease, and without any other symptom
of stomach-affection, as indicating eme-
tics, we beg to dissent from the practice.
The patient was now bled and purged.
The blood was inflamed. The venesection
was repeated, and proper remedies, under
existing circumstances, appear to have
been judiciously administered. In two-
or three days, the symptoms being nearly
the same, Dr. S. hazarded a diagnosis,
that there was an aneurismal tumour, or
a tumour formed by enlarged glands, at
the origin of the bronchi, especially in
the left side. As the diagnosis turned
out to be wrong, on both points, we beg
to offer theable author our humble tribute
of praise for this candid and laudable con-
fession. If all medical men, in all ages,
but especially in this age, had followed,
and did follow, Dr Seymour's example,
medicine would be a great gainer. We
need not pursue the details. The ex-,
pectoration became profuse?the breath-
ing difficult?the pulse unequal in the
two arms, and he died suddenly on the
2d December, sixteen days after Dr. S.
was called in. We shall give the dis-^
section in the author's own words.
" The examination was made by Mr.
Caesar Hawkins, in the presence of Dr.
P. Latham, Mr. Stone, and myself.
"A tumour was found in front of the
chest, above the heart, situated, for the
most part, anterior to the roots of the
lungs, but surrounding the lower part of
the trachea and both bronchi, the trunk
and branches of the pulmonary artery,
the pulmonary veins of the left side, the
arch of the aorta and left carotid and
subclavian arteries, but leaving the an-
terior part of the ascending aorta and
arteria innominata uncovered. Behind
it was in contact with the oesophagus,
and below with the upper part and left
* Though Dr. S. does not deign to
mention the stethoscope, we defy him or
any man to put down the above diagnos-
tic particular, except through the medium
of auscultation. Dr. S., is therefore, a
self-convicted auscultator.
488 Periscope ; on, Circumspective Review. [Feb. 9
side of the pericardium; thence it ex-
tended into the left lung, so that nearly
half of that viscus was occupied with a
similar congeries of globular tubercles,
the largest about two inches and a half
in diameter. The tumour of the lung
adhered to the left side of the peri-
cardium, to the diaphragm, and to the
vertebrae and heads of the ribs behind,
so that the lung could not be removed
without tearing through the tumour.
Most of the masses composing the tu-
mour were of a white colour, but some
were black in the centre, and others had
begun to become soft and red in the
inside. Where the tumour was in con-
tact with the diaphragm, that membrane
had become softer. A rupture had taken
place, by which an effusion of blood had
occurred, from the centre of one of the
tubercles behind the vena cava descen-
dens into the cavity of the pericardium,
which contained about a pint of fluid
blood. The back part of the aorta had
also begun to change in texture, though
the alteration did not yet reach its inner
coat. The calibre of the superior cava
was much lessened by the pressure of the
tumour, and in one part its coat had been
absorbed, so that a small fungous pro-
jection had taken place in its interior.
. " About an inch and a half of the oeso-
phagus nearest to the tumour had also
become thickened and contracted, the
change appearing most distinct in the
muscular tunic.
" The heart itself was healthy, but the
cavities of one of the left pulmonary
veins was greatly diminished by the
growth of the tumour, which had not,
however, affected its coats.
" The left bronchus and its branches
were much lessened in diameter by thick-
ening and pressure, and the remaining
part of the lung of the left side scarcely
crepitated being filled with mucus, and
watery exhalation. The tumour had
grown most in the lower lobe, so that
very little of the texture remained, which,
however, was solid. The pleura covering
this lobe was much thickened, and ad-
hered to the ribs. Many irregular white
masses of condensed cellular membrane
extended from the tumour into the outer
part of the lung, thicker and less liga-
mentous than the bands usually are in
cancerous tumours.
?/ The disease did not reach beyond the
root of the right lung, which was red
and full of fluid, and the pleura con-
tained a number of white spots of a
cartilaginous consistence, which were of
the size of a split pea.
" A tumour similar to that in the chest,
and about the size of a small orange, was
situated above the head of the pancreas.
"The rest of the viscera were healthy."
The disease was the fungus hsema-
todes?the medullary fungus of English
writers?the " Encephaloides des Pou-
mons"of Laennec, the cancerous tubercle
of Bayle and others. We repeat it, that we
are exceedingly glad to rank Dr. Seymour
among the auscultators of the present
day?as we are sure the zeal and talents
of that physician will advance the study,
as well by his own exertions as the ex-
ample which he will set to the students
of this metropolis.
7. Redness op the inner Surface op
Blood-vessels.
As this phenomenon leads many people
astray, in their speculations as to its
causes and effects, we shall take this op-
portunity of stating the sentiments of
Laennec on the subject, as they are en-
tirely in consonance with our own obser-
vations.
Corvisart noticed this redness* but
avowed his ignorance of its nature and
cause.' Frank observed it throughout the
whole courser of the arterial system, and
concluded that it was the cause of a pe-
culiar and uniformly fatal fever! The
same opinion has been adopted by Krey-
sig, Bertin, and Bouillaud. But, let it
be remembered, that mere redness of a
part naturally white, does not authorize
us to pronounce it in a state of inflamma-
tion. The phenomenon is frequently seen
on the inside of the aorta and pulmonary
artery. The colouring is of two kinds,
scarlet, and a violet hue. Sometimes
the redness is confined to the inner mem-
brane?at other times, it penetrates the
fibrous, and even the cellular texture.
The colour is quite uniform, as if painted,
and without any trace of vascularity.
Sometimes this stain diminishes pro-
gressively from the origin of the aorta;
but frequently it terminates abruptly,
with irregular edges. In the midst of a,
1828] On a peculiar Disease of the Cranial Bones. 489
Very red portion, we sometimes see a
circumscribed spot of the natural white
colour. When the aorta contains very
little blood, the redness only exists in
the part in contact with this fluid. The
surface of the sigmoid and mitral valves
sometimes exhibits this phenemenon?
occasionally the whole of the arterial sys-
tem. Sometimes the auricles and ven-
tricles alone are tinged?and then it is
observed that the heart is full of blood,
and the arteries nearly empty. The red-
ness is attended by no appreciable thick-
ening of the membrane, and entirely dis-
appears after a few hours' maceration.
Laennec is very doubtful whether this
redness ever gives rise to any general
symptoms, so constant or severe as to
indicate its presence. For our parts, we
have no doubt on the subject. Like him
"We have found it in bodies dead of dif-
ferent affections, and without any symp-
tom, during life, that could lead to a
Suspicion of its existence.
M. Laennec has seen the violet hue of
the membrane in subjects dead of putrid
fevers, emphysema of the lungs, and dis-
eases of the heart. All these individuals
had remained long in a moribund state,
with suffocation. In all, the blood was
very fluid, evidently altered, with signs
of premature decomposition in the body.
Accordingly, we more frequently observe
this phenomenon in Summer than in Win-
ter. Both kinds of redness?especially
the violet, are accompanied by more or
less softening of the heart, and an in-
creased humidity of the arteVial tunics?
the consequence, most probably, of putre-
faction.
Upon the whole, we may safely con-
clude that this tincture of the internal
surface of arteries is owing either to some
morbid change in the blood itself, or, at
all events, to some process which takes
place in articulo mortis, or post mortem.
A remarkable instance lately occurred in
St. George's Hospital, in the case of a
man who died of phlebitis. The whole
arterial system was tinged.
S. Peculiar Affections of the Cra-
nial Bones.
Dr. Abercrombie has dedicated a section
his late work to certain affections of the
bones of the cranium, to the investigation
of which he was led by the following case.
A female, 48 years of age, fell down
stairs, and received several contusions,
one of which, on the head, confined her
for some days. From this time, her health
was bad?she had fixed pain in the head,
and disordered stomach and bowels. Still
she attended to her domestic concerns
till within three weeks of her death, when
she was seized with fever and high deli-
rium. These subsided after venesection,
and next day erysipelas appeared on the
face, which, also, went off. She recruited
a little, and was able to leave her bed,
but complained of fixed and deep-seated
pain in the right side of the head, a little
above the ear, from which there was some
discharge. Three days before death (one
year from the date of accident) she be-?
came comatose, with partial paralysis of
the left side.
Dissection. The bones of the cranium
were very soft. The inner surface of the
skull-cap, when raised, exhibited a sin-
gular state of disease. The inner table
seemed to be wanting through its whole
extent, and there appeared the rough,
irregular, and cancellated structure of the
central part of the bone. Between this
surface and the dura mater, there was a
deposition of soft adventitious membrane
of a yellowish colour, about the tenth of
an inch in thickness. On raising the
skull-cap, this membrane was found to
adhere to the dura mater, leaving exposed
the irregular cancellated structure of the
bone. In other places it adhered to the
bone, exposing the dura mater of its na-
tural appearance. The greatest degree
of erosion was in the parietal bones,
where several portions were thin and
transparent?in. a few parts perforated.
The external surface of the cranium was
of a natural appearance. In the lower
part of the right hemisphere of the brain
there was an extensive abscess. On the
petrous portion of the right temporal
bone, the dura mater was of a dark co-
lour, and detached from the bone, which,
last was sound.
There was no reason to suppose any
syphilitic taint in this woman's constitu-
tion, the cranial affection must, therefore,
have been the result of slow inflammatory-
action following the blow, and gradually
destroying the bone by caries. Dr. A.
has not been able to find any case 011 re-.
490 Periscope ; or, Circumspective Review. [Feb. 9
cord precisely similar to the foregoing.
Desault mentions a case, where death
followed a blow on the head. The bone
appeared sound externally, but the inter-
rial table was blackened throughout the
whole extent of one of the parietal bones.
The dura mater adhered to the parietal
bone as firmly as to the other parts of
the cranium, and there was suppuration
on the surface of the brain. Some other
cases, analogous to this last, are collected
from surgical writers by Dr. A. who con-
siders them as examples of an uncommon
modification of disease of the cranial
bones, confined principally to the inner
table. The more common modification,
however, is that which occurs in the outer
table, or which affects the whole depth
of the bone, with the history of which
disease some remarkable phenomena are
connected. It seems to be a low inflam-
mation, which may arise from injuries
extremely slight?or without any obvious
cause. Its progress is very slow, and it
may terminate by exfoliation of a part
of the outer table, or it may affect the
whole bone, and, extending to the brain,
prove fatal. Mr. O'Halioran mentions
the case of a man who was seized, with-
out any injury, with pain in the upper
part of the os frontis, which increased in
violence, and unfitted him for his avoca-
tions. In the course of four months an
abscess formed and burst?the bone was
found carious and perforated by an open-
ing, through which the dura mater was
seen covered with pus. The piece of
bone became loose, and separated in ten
days. The patient recovered. Dr. Aber-
erombie does not think that the trephine
promises much success in these cases.
We think differently, and shall shew
cause in our next Fasciculus.
9. Irish Col.ij2gk of Physicians.
We are glad to see our velocipede cotem-
poraries, of all colours, taking so much
interest in the medical politics of the
jSister Island. It appears that the Irish
College has determined to admit licen-
tiates without a university degree. The
only essential conditions are?"four years'
study?certificates of attendance on the
professors of the school of physic, and
the professor of midwifery?two years
attendance at any chartered hospital?
an examination."
The Medical Gazette has strongly ridi-
culed, and, of course, censured, this new
regulation. For our own parts, we do
not find fault with it. If there is to be a
certain degrading title kept up among
physicians, namely, that of licentiate,
why, in the name of sense or science,
make the acquisition of a diploma from
a regular university, an essential item in
the list of qualifications ? It is quite suf-
ficient that a man who has studied his
profession for four years, should be ob-
liged to have branded on his forehead the
term licentiate, before he can practise,
without being also obliged to degrade a
university, as well as himself, by passing
under the yoke. If the examination, in
the Dublin College, be properly con-
ducted, and the examiners take care to
see that the proofs of four years' study
be authentic, then we say that the said
regulation must ensure properly qualified
practitioners for the public?and that is
the main object of all medical institu-
tions. This license does not, we sup-
pose, confer the doctorate upon the in-
dividual, but merely gives him permission
to practise. While we decidedly object
to the obnoxious term of licentiate,
enjoyed by every pedlar who sells ribbons
through the country, and every hawker
who goes bawling about the streets with
newspapers and penny pamphlets, we
cannot see any mortal offence in the prin-
ciple of the regulation. It would, indeed,
be a fortunate and happy circumstance,
if the same regulation could be extended
to medical practitioners of all kinds.
10. Prize Essay and Discussion on
Tubercles.
The Royal Academy of Medicine offered
last year a prize for the best dissertation
on the subject of tubercles, and, at a late
sitting, M. Rullier read a report of the
commission appointed to examine the
candidate essays. The motto of the first
essay was " cujusvis est hominis err are."
The author passes in review the clashing
opinions entertained by pathologists as
to the origin of tubercles. Are these
bodies (as some represent them) acci-
dental tissues developed in certain parts
1828]
On Tubercles. 491
hy a kind of anomaly of nutrition?or
are they the product of a morbid secretion,
a species of concrete pus disposed in the
areolae of parts ? The author cannot tell.
Another, and perhaps a more interesting
question is, how are these bodies resolved?
The author thinks there are three modes
employed by Nature?softening, atrophy,
and resorption. Messrs. Thenard and
Dulong having found in tubercles the
Same salts, and in the same proportion,
in bones (phosphate and carbonate of
1'me) the author attributes the formation
?f these bodies to a deviation of the above
salts from the bones. He strengthens this
hypothesis by the fact of the greater light-
ness of the bones of tuberculous subjects,
and the exuberant proportion of phos-
phate of lime in the milk of cows affected
with tubercles. The commission attaches
great importance to this theory. After
alluding to the fact, that these bodies are
found in the foetus, he observes that
hitherto they have been discovered only in
themammiferous animals and inbirds. In
t'le first class, they are seen almost exclu-
sively in the herbivorae. It is certain
that tubercles have never been detected
in dogs. In respect to the effects of
tubercles in the organs where they exist,
it appears that these effects are nearly
null till the tubercles begin to soften.
Then the circumjacent parts inflame,
ulcerate, and form accidental tissues des-
tined, he thinks, to isolate and envelope
them. As to the primitive seat of these
bodies, the author concludes that it is
neither in the lymphatic system, nor in
the mucous follicles, but in the cellular
tissue. Tubercles in the lungs, according
to the author, are the sole cause of phthi-
sis, notwithstanding what Bayle and
others have said to the contrary. The
commission think this assertion is too
absolute, and admit the existence of can-
cerous, calculous, melanic, and even os-
seous phthisis. The commission, also,
hlames the author for having attributed
too much influence to pulmonic inflam-
mation, in the production of tubercles.
They think that these bodies exist be-
fore the phlegmasia?or if they become
developed afterwards, it is owing to the
cachectic habit of body produced by the
Preceding inflammation. The commis-
s'on ridicules the assertion of Laennec,
that tuberculous excavations have healed
?or, m other words, that phthisis has
been cured. Speaking of bronchial
phthisis, where the softened tubercles
have discharged themselves by fistulous
openings into the oesophagus or bronchia,
he notices the curious fact, and illustrates
it by a preparation, that the perforation
is always through one of the cartilaginous
rings, and never through the intermediate
membrane. He remarks that tubercles
of the mucous membrane are very gene-
rally confined to the sub-diaphragmatic
portion of the alimentary canal-?and
especially to the lower portion of ileum,
where they lead to ulceration. In laryn-
geal phthisis, he maintains that tubercles
in the larynx are the primary source of
the disease?an opinion which the com-
mission contests. They believe that ul-
ceration is the primary condition.
In respect to tubercles in the brain, the
author notes the intermissions of the
nervous disturbances dependent on these
tubercles?a fact that is truly inexpli-
cable. It certainly strengthens the doc-
trine, that a pure intermittent fever itself
may depend on some lesion of structure,
notwithstanding the periodical states of
apyrexia. The author thinks that tuber-
cles in the bones, a disease which has
excited but little attention, are the cause
of many cases of white swelling, spon-
taneous luxations, vertebral caries. He
shewed a preparation exhibiting a veri-
table tuberculous cavern in the spinal
column. The commission passed high
encomium on the Essay, but did not ad-
judge to it the prize. They awarded its
author, however, a medal, value 500
francs.
The reading of this report led to an
animated discussion. M. Andral freely
censured the doubts cast by the com-
mission on the possibility of cicatrization
in tuberculous excavations?and conse-
quently the sanability of phthisis. Both
these are proved, he observed, by symp-
toms, by pathological anatomy, and by
analogy. Thus, a person will present all
the symptoms of phthisis, with unequi-
vocal pectoriloquism under one of the
clavicles. The patient recovers, and dies
of some other disease ; and in the place
where the pectoriloquism was heard, will
be found a cavity lined, or filled, or filling
up with various kinds of tissue, proving
the restorative process that had been
going on or completed. Analogy shews
us that the. excavations of diseased lyui-
492 Periscope ; on, Circumspective Review. [Feb. 9
phatic glands will fill up in various ways.
M. CHOMELalso criticised the commission
on this point. They admitted the possi-
bility of cicatrization in cases of insu-
lated tubercles?and this was all that
Laennec and others contended for. No
man ever stated, that a tuberculated lung
would recover after several excavations
had formed. This physician adverted to
the intermissions of functional disturbance
in organic diseases. He conceived that,
in such cases, the symptoms of functional
disorder were not exclusively dependent
on the structural lesion (which is perma-
nent of course) but upon some accidental
and temporary causes, which unite their
influence with the organic disease. M.
Ruilier replied and explained, by which
explanation, it appeared that he enter-
tained nearly the same opinion as Laennec
and others, respecting the cicatrization of
tuberculous excavations.
The intermission of symptoms in or-
ganic diseases, however, led to some
sharp controversies?but the problem
was not solved by the " collective wis-
dom" of the Academy. On breaking the
seal of the motto, the author's name
was found to be M. Larcher, internal
eleve of the Maison de Sante, rue
Fnubourgh St. Dennis.
HOSPITAL PRACTICE.
1. LA CHARITE.
Remarkable Disease of the Mucous
Membrane of tiie Bladder. By M.
Louis.
This distinguished pathologist remarks
that, of all other mucous membranes, that
of the bladder is the least frequently found
diseased. This is the more to be won-
dered at, when we consider the great va-
riety of alterations to which the urine is
subject, from diseases and from diet. M.
Louis examined the mucous membrane
of the bladder in 500 subjects, dead of
various diseases, and found six instances
where this surface was affected with sim-
ple redness or injection of vessels, but
without any softening of structure. In
two or three other cases, there were sof-
tening and other species of organic lesion.
Two cases we shall present in an abridged
form to our readers.
Case 1. A man, aged 77 years, was
brought to the hospital, on the 23d March,
1827, in a desperate condition. It was
learnt that, lately, he had been obliged to
make water very frequently, but without
pain. Two or three times he passed blood
in his urine in considerable quantities?
and, for a fortnight before he entered the
hospital, his water contained more or less
blood. He died the next night, presen-
ting gangrene of the lower extremities,
&c.
Passing over the morbid appearances
in other parts of the body, we find that
the bladder was contracted to about the
size of a man's fist, and was slightly pro-
minent above the pubes, being adherent
to the transverse arch of the colon by a
membranous cord, of about two inches
in length. It contained three or four
ounces of dark-coloured, purulent liquid.
The internal surface of this receptacle
was red, and presented an adventitious
tissue, better than a line in thickness,
and of a filamentous structure, but quite
soft and easily lacerable. The urethra
was perfectly sound. M. Louis has not
been able to find a case of morbid ana-
tomy of the bladder, precisely similar to
the above, in any of our pathological wri-
ters. There can be no doubt, however,
that it was a product of inflammation?
and, moreover, that it was rather a mor-
bid development of the mucous mem-
brane itself than a new or adventitious
structure formed by disease.
This patient died of a rupture of the
left auricle of the heart, within the peri-
cardium?an extremely rare accident.
Case 2. A female, aged 45 years, was
received into La Charite, on the 15th
March, 1827, stating that she had been
ill about eight months. In the beginning
of that period, she had some uterine
discharges, accompanied by pains in
the hypogastrium and loins, which she
characterized as lancinating and tear-
ing, and with hardly any remissions.
1828] Hospital Practice. 493
The right thigh had lately become the
Seat of a disagreeable sensation of formi-
cation. The urine was passed very fre-
quently, and in small quantities at a time,
during the preceding four months. The
bovvels were constipated?the disease had
teen left to itself. On examination, there
Was perceived a swelling in the hypogas-
trium, rising a couple of inches above the
Pubes, and accompanied by the most
acute sensibility on pressure. There was
some inconsiderable discharge from the
Vagina. No fever; but the vital powers
Were evidently sinking. She lingered
out till the 28th of April, when she died.
Latterly she had been affected with diar-
rhoea.
Dissection. Omitting the notice of
some unimportant lesions in the stomach
and bowels, we come to the urinary or-
gans. The kidneys were pale, and not
more than half their natural size, and yet
their pelves and infundibula were very
much enlarged. The lining membrane
of these last parts, as well as that of the
ureters, was thrice its natural thickness.
The bladder was very small. The internal
surface presented a strange medley of
morbid productions, which it would be
very difficult to describe. There were
three layers, as it were, of diseased
growths?one appeared to consist of py-
riform vesicles, demi-transparent, con-
taining a clear, but yellowish fluid. These
were mixed with, or joined to, another
set of bodies, bearing more the charater
of tubercular bodies. The mucous mem-
brane, and the submucous tissue, was in
a state of great disease. The uterus pre-
sented several scirrhous masses growing
about its cervix and body.?Repertoire.
The above are curious specimens of
morbid anatomy.
We have said that rupture of the left
auricle of the heart, within the pericar-
dium, is a rare accident. We lately saw,
in the possession of Dr. Somerville, jun.
a fine specimen of aneurismal pouch, of
aortic origin, but within the pericardium
?that is, behind the semilunar valves.
It had attained the size of a large goose-
egg?and, ultimately, burst into the ca-
vity of the pericardium, causing imme-
diate death, of course.
2. HOPITAL DES ENFANS MALADES,
Peripneusiony of Children.
In the Hopital des Entfans Malades,
M. Guersent, has lately been induced to
try the efficacy of tartarized antimony,
in the pulmonic inllammations of chil-
dren, in the above institution, and has
published, in the September Number of
the Archives Generales, a few cases
illustrative of this plan of treatment M.
Guersent, does not, like Laennec, and
some of the Italian physicians, trust,
almost exclusively, to antimony. He
bleeds vigorously at first?but finding
that this measure is insufficient, he em-
ploys the above remedy in large doses.
Thus, in the first case, a boy of 12 years
of age?the inflammation occupied al-
most the whole of the left lung, and the
second stage, or hepatization, had taken
place. The patient had been repeatedly
bled and leeched, but still the cough, the
dyspnoea, the fever, the viscid and scanty
expectoration, threatened the boy's life.
In this state, M. Guersent ordered six
grains of tartar emetic to be dissolved in
twelve ounces of orange-flower water,
and this portion to be taken in divided
doses, every two or three hours, in the
course of one day and night. The first
dose vomited the child once, and pro-
duced three or four alvine evacuations.
The three next doses occasioned neither
sickness nor purging. The little patient
had some sleep, and next morning the
symptoms were mitigated. During the
succeeding two days, the same medicine
was administered, without any inconve-
nience, though the daily quantum was
augmented to eight grains. The disease
was very quickly subdued. Some of the
other cases were more formidable, and
the same treatment was adopted with
success.?Archives.
3. WEATH HOSPITAL, DUBLIN.
Hepatic Disease. Drs. Graves and
Stokes.
In the following case, related by Drs.
Graves and Stokes, from the Meath Hos-
pital, some observations will be found,
corroborating certain opinions which we
gave in our review of Dr. Blight's work,
494 Periscope; on, Circumspective Review. [Feb. 9
in the second Fasciculus of this Number,
page 319.
Case. J. M'CIusky, aged 11 years,
of scrofulous habit, labours under con-
siderable swelling of the abdomen, with
fluctuation and tympanitis. " The right
liypochondrium appears rather full; upon
examination, the liver can be felt extend-
ing across the left hypochondrium, and
as far down as the umbilicus, presenting
a defined edge." He had oedema of the
left leg?no cough or dyspnoea?good
appetite?much thirst?urine copious,
light-coloured?and depositing albumen
in abundance when heated. Bowels are
regular, the pulse 125, tongue clean and
moist, sleeps well. The complaint was
of twelve months standing. One grain
of calomel was ordered to be taken every
night, and some mercurial frictions to
the right hypochondrium. On the eighth
day of this treatment, the mouth was
affected, and the calomel was omitted.
Having caught cold, a smart ophthalmia
occurred, which required leeching, &c.
Afterwards, as the swelling of the abdo-
men continued, the calomel was renewed,
in two grain doses, and half a drachm of
the spir. a;th. nitr. was given twice a day.
The belly became reduced in size?the
urine more copious, but still coagulable
by heat. In a short time afterwards, he
was discharged, the abdomen being nearly
reduced to its natural size, and the appe-
tite good. The following extract from
the observations appended to this case,
we deem worthy of insertion.
" It is not easy to determine the nature
of the hepatic tumour which was so very
considerable in this boy. It was slow in
its increase, and not attended with well
marked symptoms of chronic inflam-
mation of the liver. There was no ten-
derness on pressure, nor any pain or
uneasiness in the right hypochondrium.
There was no evident derangement of the
biliary secretion, and his appetite, sleep,
strength, and nutrition, were scarcely
impaired. On the other hand, the oedema
of the left leg, the commencing ascites
and tympanitis, the albuminous urine,
accelerated pulse and increased thirst, all
united to prove that the constitution had
begun to suffer in consequence of the
diseased state of the liver. The alter-
ative doses of mercury, which were cau-
tiously exhibited, were evidently pro-
ductive of much benefit. The mixture
with spirit of nitrous aether seemed useful
in relieving the Tympanitis. In this,
and many other cases, ivliere considerable
organic alteration had taken place in the
liver, ive have observed an apparently
healthy secretion of bile. Thus we have
found bile of an healthy colour and con-
sistence in the gall bladder, when the
substance of the liver ivas taberculated
throughout. Naturally coloured alvine
discharges therefore furnish no proof that
extensive organic disease of the liver docs
not exist."
" Concerning the albuminous state of
the urine we may remark, that it is no
proof of an inflammatory condition of
the constitution, it merely indicates con-
siderable disorder of the function of assi-
milation. In health, a certain portion of
animal matter is contained in the urine,
in the form of that highly animalized
substance, urea. This may be increased
so much in quantity above the healthy
standard as to constitute a disease. When
the assimilative powers are more de-
ranged, the animal matter of the urine
ceases to assume the more highly ani-
malized form of urea, and is voided in the
form of albumen, which contains much
less nitrogen than urea. In a state of
the system still further depraved, it passes
off in the form of sugar, which contains
no nitrogen, and is the least highly ani-
malized. In diabetes, it is probable, that
the urea is voided in increased quantity
at first; as the disease proceeds the ani-
mal matter is voided in the shape of albu-
men, and afterwards of sugar. When
diabetic patients are getting better, then
the contrary seems to take place ; and
when the sugar diminishes the albumen
increases or reappears, and afterwards is
replaced by the more healthy secretion of
urea. Doctor Prout was the first to es-
tablish the existence of these three dif-
ferent species, or rather stages of diabetes.
In dropsy the appearance of albumen in
the urine is a bad sign, as indicating a
depraved assimilation and a source of de-
bility. We have established, by numerous
experiments, that when there is much al-
bumen, there is scarcely any urea in the
urine, and vice versa, or more generally
that the proportion of urea is inversely
as that of the albumen. How far the
treatment suited to diabetes may be also
applicable to cases of chronic dropsy with
^828] Hospital Practice.
495
albuminous urine, and unattended by
0l'ganic disease, experience alone can
decide."
The passage which we have marked in
'talics, is that to which we wish to draw
the attention of Dr. Bright, and the
Public. But the whole of the observations
?f our intelligent authors are deserving of
Notice.
Pathology of Fever. Drs. Graves
and Stokes.
We believe that none, even of our conti-
nental brethren, have taken greater pains
to investigate the causes, the seat and the
consequences of fever, than the two phy-
sicians above-named. Their sentiments,
therefore, on the pathology of this wide
spread disease must be interesting to the
profession at large. These sentiments
may be gathered from some observations
appended to a case of fever (among many
others) treated in the Meath Hospital.
" The epigastric tenderness, with nau-
sea and vomiting, so common in this
fever, seemed to be caused by inflamma-
tion of the mucous membrane of the sto-
mach. In most of the fatal cases this
Was found of a dark-red colour and very
soft, a condition evidently produced by
violent inflammation. In others the red-
ness was not so extensive, dark, or con-
tinuous ; although we acknowledge, that
in the present fever the above symptoms,
depending on inflammation of the sto-
mach, are very frequent, yet we have
seen some cases where there was no evi-
dence of any local inflammation what-
soever ; and in others again, have ob-
served that some other organ, as the brain
or lungs, was the seat of inflammation,
while the stomach was free. We cannot
subscribe therefore to the opinion, which
supposes a local inflammation to be the
root of all fevers, or to that which attri-
butes their origin solely to inflammation
of the mucous membrane of the stomach
and alimentary canal. In our dissections
we have in some cases found the brain
inflamed, in more cases the lungs, and
still more some part of the digestive or-
gans. We do not recollect to have found
in one instance, out of very numerous
dissections, a fatal case of fever which
did not exhibit some serious local lesion
of an inflammatory nature; so that while
we deny, from our observation of cases
during life, that fever necessarily implies
local inflammation, dissection has con-
vinced us that the occurrence of local
inflammation during fever, is the general
cause of its fatal termination. Let us
here observe that the latter inference is
by no means contradictory of the former;
for in the fatal cases, accurate observa-
tion always detected, (hiring life, the seat
of the inflammation ; so that in those
cases which terminated favourably, and
where no such symptoms existed, our
inference that no local inflammation had
been present, receives additional strength
from our post mortem examinations, for
there we always found inflammation where
we expected to find it; that is to say,
we were always able to pronounce on its
situation, so far as to tell, before the body
was opened, hi ivhich cavity the inflamma-
tion would be found. The post mortem
examinations have been always conducted
by ourselves with the greatest care, and
we generally spend between two and three
hours in the examination of the body,
being convinced that nothing has con-
tributed to retard the advancement of
medicine so much as superficial post mor-
tem examinations. In examining the ab-
domen, we first note the general appear-
ances of the intestines, and then take out
the whole intestinal tube, which we slit
up with an enterotome at its mesenteric
attachment; this is done in order to
avoid dividing any of the follicular patches
or ulcerations in the small intestines,
which are always situated at some dis-
tance from the mesenteric attachment.
During this process we examine the con-
tents of the alimentary canal; and after-
wards having first carefully washed the
mucous membrane, we remark its ap-
pearance throughout its whole extent
from the stomach to the rectum. The
morbid anatomy of the brain, the lungs,
and the intestinal canal, has, within these
very few years, received so many impor-
tant additions, so much light has been
thrown on this subject by more accurate
investigations, that we would hesitate
much in drawing any conclusions from
the dissections of fever subjects recorded
before this period. Indeed, we could
prove that in most of even modern works
on the pathology of fever, morbid appear-
ances have been frequently mistaken, and
more especially that many things, both
496 Periscope; or, Circumspective Review. [Feb. 9
in the brain, lungs, and alimentary canal,
have been set down as morbid, which
really are not so ; consequently, conclu-
sions, not at all justified by the state of
the parts, have been drawn. Thus, we
hear of sanguineous congestion in the
head, and morbid vascularity of the brain,
intestines, &c. where the very accounts1
given contain internal evidence, that these
supposed morbid appearances had either
no claim to that appellation, or resul-
ted from changes which took place imme-
diately before or after death. In fact,
we look upon the morbid anatomy of
fever as a subject which requires to be
investigated almost de novo. Our asser-
tion, that we have hitherto found evident
lesions of vital organs in all the fever
subjects we have dissected, is, we are
aware, opposed to the recorded experi-
ence of many authors, who relate nume-
rous cases in which no morbid alteration
of any consequence could be detected.
We question however very much the ac-
curacy of such dissections, for, as has
been well observed by Rostan, nothing
is easier than to find nothing. We doubt
whether such persons have injured medi-
cal science more than those who have
found too much."
We recommend the above careful mode
of post mortem researches to the atten-
tion of our brethren in this country?
especially those who have the superin-
tendance of public institutions, and who,
consequently, have less difficulties to con-
tend with, than those in private practice.
We believe that very few cases of fever
go on to a fatal termination, without the
occurrence of organic changes?at least
of inflammation in some of the great vis-
cera. These may be the cause of death,
but not of the fever.
4. ROYAL INFIRMARY OP EDIN-
BURGH.
In a paper on bronchotomy, Dr. Cullen
has related the following interesting case
that occurred some years ago, in the
Royal Infirmary.
Case? A healthy married woman, aged
25 years, having laboured under consi-
derable depression of spirits and mental
distx-ess, attempted suicide with a table-
knife, but only made a flesh-wound over
the crico-thyroid membrane, which was
slightly perforated. Through this aper-
ture the woman breathed, when Dr. Cul-
len saw her two hours afterwards. The
wound was not of much consequence in
itself, but the patient was in an unplea-
sant condition in other respects. She
was slightly delirious?dwelling on her
domestic grievances ? pulse small and
feeble?face flushed?tongue white and
tremulous ? countenance expressive of
anxiety and suffering. The wound was
dressed?the head shaved?and ice ap-
plied to the scalp. Some wine and water
was given to her, when she was disposed
to sink?a cathartic was administered,
and she was confined in a dark room.
She passed a quiet but sleepless night,
and next day had cough. Delirium re-
turned in the evening. Leeches to the
temples and forehead. Third day. Rather
better?cough, with difficult expectora-
tion. In the evening, the stethoscope
detected inflammation in the smaller
branches of the bronchia. A large blis-
ter to the sternum. .Fourth day. Deli-
rious, probably from agitation at seeing
her husband. Fifth morning, early, she
was reported to be dying. Dr. Lubbock,
the house-surgeon, found her on the point
of suffocation, with death-rattle in the
throat?livid face?cold extremities. Dr.
L. quickly introduced a large curved ca-
nula into the wound, which enabled the
patient to take a deep inspiration, and
the breathing immediately became easier.
She now brought up a quantity of mucus
through the canula by a process which
Dr. C. will not allow to be exactly cough-
ing. The alarming symptoms soon sub-
sided, and the expectoration became free.
The same train of dangerous symptoms
again occurred in the evening from the
slipping out of the canula, but were again
relieved by closing the opening in the
larynx, and inducing the woman to cough
up the mucus by the natural passage.
This, however, had but a temporary effect,
and she died the same night.
Dissection. The vessels of the brain
were found much injected, with consider-
able effusion in the sub-arachnoid cellular
tissue. There was no disorganization of
the substance of the brain, but there was
much serum in the ventricles and at the
base of the brain. The bronchia, espe-
cially on the left side, were obstructed
with bloody mucus?the lungs sound.
1828]
Pulsating Tumours of the Scalp. 497
The mucous membrane, in the neigh-
bourhood of the wound, was inflamed but
not thickened. There was no effusion
into the submucous cellular tissue, and,
consequently, no diminution of diameter
in the glottis. The membrane of the
trachea and bronchia was highly inflamed.
?Ed. Journal.
Dr. Cullen seems to think that this
poor woman's death was occasioned by
the state of the air-passages. But we
imagine the phenomena presented in the
brain were more likely to occasion the
fatal event. The case, however, has been
evidently brought forward to illustrate
some observations made in a preceding
number of our Northern contemporary,
on the difficulty which a patient has in
coughing up, or rather expectorating the
phlegm, after bronchotomy. Indeed, it
is probable that, had it not been for some
observations which we made on Dr. Cul-
len's paper, this last case might not have
seen the light. We said that a patient
can cough?that is, forcibly expectorate
through a tube in the trachea. Dr. C.
maintains that is not coughing, strictly
speaking. We will not quarrel with the
learned Doctor about the name of pro-
cess ; but, having seen a patient cough
for weeks and months through a canula,
We cannot give up the evidence of our
senses, even to support an ingenious hy-
pothesis. If Dr. Cullen will look into
the 5th volume of the Medico-Chirurgical
Journal and Review, for January, 1818,
he will there see the case of Mr. Price,
of Portsmouth, who, to this day, breathes
through the tube. Dr. C. will see the
diurnal details of the case, where the act
of coughing is every day stated?and
where, on the 55th day after the opera-
tion, the following passage occurs :?
" Last night he felt a piece of bone (ossi-
fication of the thyroid cartilage) fall down
into the lungs, and has ever since been
in a dreadful state of coughing. He feels
the piece come up to the tube, but, as he
cannot get it out, it falls back, and keeps
him in constant agitation." A large ca-
nula was quickly constructed, and forced
into the aperture, and, " shortly after-
Wards, in a violent convulsive cough, he
threw out the piece of bone (No. 1) quite
across the room where he was sitting."
P. 6. Now we think Dr. Cullen will
absolve us from the charge of forging a
case ten years ago, and putting down
Vol. VIII. No. 16.
expressions that were to contradict his
theory at the present time. After this
document, we leave him to persuade the
world, by ivords, that a man cannot cough
after a tube has been introduced into the
trachea.
S. GLASGOW INFIRMARY? HOSPITAL
OF SURGERY, &C.
Pulsating Tumours of the Scalp.
The first case which we shall notice,
occurred to Dr. Maclachlan, one of the
surgeons to the Glasgow Royal Infir-
mary, and is related by that gentleman,
in the first number of the Glasgow Medi-
cal Journal.
The patient, a discharged soldier, ast.
30, applied to Dr. M. with a tumour on
the left side of the scalp, presenting the
following appearances:
" Soft, puffy, pulsating, and somewhat
elastic swellings of a varicose appear-
ance were found to occupy the course of
the temporal, posterior auris, and occi-
pital arteries and their principal branches;
each branch terminating by a tortuous
extremity. These swellings could be made
partly to disappear on pressure, but on
its removal, they speedily regained their
former volume. They pulsated through-
out their whole extent, and the pulsations
were synchronous with those of the heart.
By pressing on the common carotid, the
pulsations ceased all along the swellings;
and, by intercepting the flow of blood
through the temporal or posterior auris,
the throb \vas interrupted in correspond-
ing parts of the tumour. They were not
painful on being handled, but he com-
plained much of the torture he had ex-
perienced for the last two months, from
the throbbing, which often deprived hiin
of rest for nights together, and, as he
said, made his existence miserable to him.
The integuments covering the swellings
were of their natural colour; only at those
points which were most prominent, they
had a slightly blueish-red tinge.
" This arborescent tumour commenced
in front of the ear, immediately over the
zygoma, and quickly swelling out, it be-
came of the size of a split lemon, lying
transversely over the ear. It sent a pro-
cess forwards on the forehead, communi-
cating by a tortuous extremity with the
31
498 Periscope; or, Circumspective Review. [Feb. 9
supra-orbitary twig from the internal ca-
rotid ?, a large process upwards to the
crown of the head ; and backwards, the
main body of the tumour communicated
with the puffy swellings of the posterior
auris and occipitalis, which latter vessels
gave a varicose feeling to the scalp over
the left side of the occiput.
" The largest and most prominent
part of the tumour was immediately over
the ear ; at this point, the throbbing was
very violent, and the integuments being
very thin and rather pointing, it threat-
ened ere long to burst."
Ten years previously the temporal ar-
tery had been opened, and a small aneu-
rism formed upon it. For this the vessel
was cut across, but without success, and
a ligature was then applied. The little
tumour disappeared for a time, but after-
wards returned, though for the first five
years it gave him little uneasiness. Pres-
sure had been already employed, and the
patient would not again submit to it,
but wished his carotid artery to be tied.
Dr. M. however, proposed taking up the
vessels separately that fed the tumour,
and, if this should fail, tying the common
carotid. Accordingly, assisted by Pro-
fessor Towers and Dr. Anderson, he ex-
posed the temporal artery, as it emerges
from under the parotid, and found the
vessel larger than a goose-quill, thinner
and more diaphanous in its coats than a
vein, and pulsating with much violence.
A ligature was applied upon the vessel,
with a compress and bandage for addi-
tional security. Pulsation ceased in the
anterior and central parts of the tumour,
which felt flaccid and doughy, but, Dr. M.
not liking the state of the vessel, deter-
mined on securing the carotid, which was
done next day in the presence of Profes-
sors Burns and Towers, Drs. King and
Anderson. The steps of the operation,
which was performed on the 10th July,
we need not describe, but, immediately
after its completion, the tumours of the
head felt flaccid and lost their pulsation,
although they were but little diminished
in size. Next day he was seized with
pain in the right side ; pulse 120; skin
hot. V. S. at two bleedings, to 70 oz.
Saline purges?a blister. V2th. Pain of
chest easier?breathing oppressed. Tinct,
digital.?anodyne at bed-time. 13?/j. Much
the same, but, in the afternoon, there
came on pain in the region of the liver, in-
creased on pressure. V. S. ad 3xxiv.?
castor oil?turpentine enema??anodyne.
14th. Delirious?respiration oppressed?
pulse 144, feeble. Died at 5, p. in.
Sectio cadaveris, fifty hours after death.
?In consequence of the heat of the
weather, putrefaction had made some
progress. There was some pus in the
anterior mediastinum, and about a pint
of greyish muco-purulent matter in the
right cavity of the pleura, with a little
bloody extravasation into the left. The
wound had adhered by the first intention,
but was partially opened up by putrefac-
tion. The carotid artery appeared to be
quite sound in the neck?small clots had
formed above and below the ligature, and
the artery was puckered from the recent
deposition of lymph. Its inner coats were
divided, its external entire, but below the
ligature the inner coat was of a vermilion
tint, as was that of the thoracic aorta.
The aortic arch between the heart, and
the giving off of the left carotid, was
healthy. The temporal and other branches
of the carotid in the head " had degene-
rated into dilated tubes of great thinness
and transparency," and had become elon-
gated, contorted, and convolutedon them-
selves, so as to form, by this species of
doubling, the tumours which constituted
this singular disease. Where the thinning
of the branches began, or whether the
internal branches of the carotid had suf-
fered also, giving rise to the epileptic
fits to which he had recently become sub-
ject, Dr. M. was prevented from ascer-
taining.
Dr. Maclachlan thinks, and so do we,
that there is an obvious difference be-
tween this species of pulsating tumour,
and the aneurism by anastomosis des-
cribed, by John Bell, as " a congeries of
active arteries, absorbing veins, and in-
termediate cells." Two cases are given
by Pelletan in his Clitiique Chirurgicale,
precisely tallying with this. One patient
was a girl of 18, on whom compression
was tried, but she could not bear it.
He then tied the temporal artery, and
with good effect, but the patient died of
" indigestion." Upon dissection, the
tumour was found to consist of tortuous
and dilated arteries. Boyer, Pelletan,
and others, are of opinion, that the dis-
ease is congenital, but without sufficient
grounds. Dr.M. himself opened the tem-
poral artery in the first instance, and, at
A
1328]
Pulsating Tumours of the Scalp. 499
that time, there was no trace of enlarge-
ment. We now come to the question of
treatment. In all the cases of the affec-
tion which have been published, compres-
sion has failed, on account of the pain
which it induced ; and, when we consider
the anatomy of the parts, we might be
led to expect as much. Ligature of the
temporal artery has been tried, but, al-
though it gave a temporary relief, it does
not seem ever to have effected a cure.
This, too, might have been expected ;
for, although the disease consists, in the
niain, of the convolutions of one trunk,
the temporal, still the anastomoses be-
tween it and its fellow of the opposite
side, as well as with the occipital and
even the supra-orbital artery, must always
be enough, and more than enough, to
allow of the free ingress of the blood into
the tumour. If this reasoning applies,
and we believe that it does so, to the
tying of the vessel which immediately feeds
the tumour, must it not, a fortiori, apply
to the ligature of that great trunk which
does not immediately feed it, we mean
the common carotid ? You tie the caro-
tid, you expect to obliterate its dilated
temporal branch, and why ? Is it because
a similar operation on the femoral artery
will cure a popliteal aneurism ? The ana-
logy is not a fair one. Between the point
of the ligature and the sac, there come
off in the femoral no branches of any con-
sequence. One or two of the articular
arteries may arise just above the sac, but
they are small, and, at any rate, anasto-
mose with the tibials below, so as not to
interrupt the remora in the sac itself.
The new circulation is carried on princi-
pally by the branches of the profunda
and common femoral, (we mean its long
external descending ramus,) and not by
the vessels of the artery tied.
With the carotid it is exactly the re-
verse. First, there is the division into in-
ternal and external, and, prior to this last
terminating in the internal maxillary and
temporal, there are eight branches given
off, all of them having communications,
more or less free, with each other. This
being the case, we put it boldly to any
man of common sense, whether he can
reasonably expect toobliterate an enlarge-
ment of the terminal branch of a vessel,
whilst a full and free circulation must
necessarily be kept up between that en-
largement and the ligature. The conse-
quence of tying the common carotid is
simply this, that the vessel is obliterated
nearly as high as its division into the
external and internal, but not one jot
higher, because, above this, the circulation
is brought into these vessels by their anas-
tomosis with the inferior thyroid of the
same side, and the thyroid and other
branches of the opposite, as well as by
the free inosculations of the internal ca-
rotid. In fact, the man who imagines he
can plug up the temporal artery by ail
operation on the common carotid, might
just as fondly attempt to obliterate the
vessel of the great toe, by tying the artery
at the groin!
So much for what the operation has
not accomplished, but let us look a little
at what it has. In the case detailed by
Dr. Maclachlan, there cannot be a doubt
that it was the immediate cause of the
patient's death?in the case which we are
now to detail, there can be as little doubt
that it led ultimately to the same end.
J. Nowlan, set. 22, was admitted into
Panton-square, with a pulsating swelling
on the left parietal bone, in the situation of
the posterior temporal artery. It could be
emptied by pressure, but a large commu-
nicating branch passed across the vertex
from the opposite temple, and contributed
to supply it with blood. The cranium
beneath appeared to be very considerably
absorbed, and the integument above was
stated, in the Lancet, to be blue and ready
to ulcerate, but the latter statement we
have heard denied. The temporal artery
had been secured, at least it was so sup-
posed, by Mr. Babington, but with no
effect. The day after the man's admission,
the common carotid artery was tied by
Mr. Wardrop, jn order, as the reporter
tells us " to completely arrest from almost
every channel, the supply of blood to this
rapidly increasing vascular tumour."
Much difficulty was experienced in getting
the needle round the artery, and a free
bleeding took place (so goes the report)
from two large thyroicleal veins. The
pulsation ceased, but the tumour did not
collapse, and next day a thrill was again
perceptible. On the fifth day, there was
much fever, and he was bled to syncope?
on the 7th, ha;morrhage, partly venous
and partly arterial, occurred to a consi-
derable extent, and he was again bled to
fainting. On the 9th day, he was once
more largely bled, venous haemorrhage
500 Periscope; or, Circumspective Review. [Feb. 9
had occurred daily since the operation,
the wound was not disposed to unite, and
the tumour distinctly pulsated. 11 th day.
Pain in the left eye-ball and orbit, with
deafness, drowsiness, and mental disturb-
ance, which increased on the 12th almost
to coma, with contracted pupil. On the
13th day, he was better, but the eye-ball
was found to be greatly protruding, which
was attributed, by Messrs. Lawrence and
Wardrop, to a powerful compression on
the brain, exercised by an aneurismatic
state of the vessels of the dura mater,
communicating through the skull with
the tumour on the head ! This was cer-
tainly an odd notion enough, and turned
out to be any thing but the true one.
The protrusion in the eye-ball, however,
continued to increase?the sclerotica
sloughed, and the humours were dis-
charged. The ligature came away on
the 25th, and the wound quickly healed.
On the 26th October, the case was
brought forward by Mr. Duncan, the
very intelligent house-surgeon at Panton-
square, in the Westminster Medical
Society. After detailing the case, he
requested the opinions of the members
on the treatment to be adopted, for, at
this time, the tumour was very little better
than before the performance of the ope-
ration. It would not be difficult to raise
a laugh, were we to record many of the
opinions as to the nature of the disease
expressed by gentlemen on that occasion.
Mr. Mayo proposed excision of the tu-
mour. Dr. J. Johnson recommended
circumvallating it by a circular incision,
and tying the vessels which fed it directly,
as well as those which supplied it by
anastomosis. Nothing further was heard
about the case until the 4th January,
when the poor fellow was admitted into
the Middlesex Hospital, with lumbar
abscess, and altogether in a most miserable
state. On the 21st he died. The tumour,
at this time, was large and pulsated very
strongly.
On dissection,* the posterior temporal
artery was found to divide into two
branches, one running, as usual, beneath
the skin, the other perforating the tendon
of the occipito-frontalis, and then, con-
siderably enlarged, twisting and turning
and coiling upon itself, looking, when in-
jected, as like a varicose vein, as it is pos-
sible for a vessel which has no valves, to re-
semble one which has them. On examin-
ing the neck, the common carotid was
obliterated, and converted into a cellular
chord just up to its bifurcation, whilst
the jugular vein was in nearly the same
state of degeneration from the site of the
ligature up to the division of the carotids,
from which to the base of the brain it
was plugged with coagulum. There was
much pus at the basis cranii, and around
the commissura tractuum opticorum; it
lay between the pia mater and arachnoid,
and was continued within these mem-
branes down the whole length of the
spinal chord. The cranium was some-
what grooved beneath the tumour, just
as it would be beneath a powerful muscle,
but the pericranium was entire, and the
inner surface of the bone was perfectly
smooth and equal.
We have thus given an abstract* of
this very interesting case, and it seems to
us entirely to confirm what we observed
upon Dr. Maclachlan's. It establishes
most completely the inutility of the liga-
ture upon the carotid, for that vessel was
plugged up no higher than (indeed not so
high as) its bifurcation. This is a fact
of some importance, for, although we
cannot well be accused of belonging to
that ignavum pecus, who are eternally
prating about " the wisdom of our an-
cestors," and entertain a most conscien-
tious horror of every thing which was
not known to pathologists in the good
old days of Noah, still we do think that
if any thing is carried to excess at the
present time it is the rage for tying
carotid arteries. The operations on this
vessel which have been performed of late
by Mr. Wardrop, have been most unfor-
tunate, and sorry as we should be, to
attach blame to any man who endeavours
to establish a principle in surgery, we
would yet intreat Mr. Wardrop for his
* The principal facts stated here were
given by ns upon the cover of the last
Fasciculus. We thought it better, how-
ever, to repeat them, that the case might
not be incomplete.
* The account of the case is culled
from the Lancet ; the dissection is re-
corded in the 9th Number of the Medical
Gazette, but we had also an opportunity
of inspecting the parts ourselves.
1828] Sciatica?Hydrocele. 501
?wn sake, and for the sake of science, to
pause awhile ere he repeats them. The
only two proposals which appear to us in
ftuy way applicable to this pulsating
tumour of the temporal artery, are those
Which were made by Mr. Mayo and Dr.
Johnson, to wit, excision, and circum-
vallating the tumour by an incision. The
second is certainly less formidable than
the first, and would prove quite as effec-
tual. If, as in Dr. Maclachlan's case,
the disease be too extensive to admit of
complete circumvallation, those branches
which go to, and those which go from
the tumour might still be tied, and that,
'rom their increased size, without any
great difficulty. This, after all, is in
principle, only the operation daily per-
formed upon a punctured artery, namely,
the cutting off all influx of blood into
the wounded part, by a ligature above and
below. Some questions present them-
selves upon Mr. Wardrop's case, viz. the
joss of the eye, and the obliteration of the
jugular vein, but our limits will not allow
Us to consider them fully. We think,
however, from the difficulty experienced
during the operation?from the venous
bleeding at the time, and from that which
occurred afterwards, that the needle was
Unfortunately pushed through the coats
of the vein, and part of it, at any rate,
tied with the artery. Whether the liga-
ture of both had any effect upon the eye,
is more than we can pretend to say.
6. BARTHOLOMEW'S HOSPITAL.
Sciatica cured by Moxa.
In the seventh Number of the Gazette,
there is an interesting case related from
the practice of Mr. Earle. The patient
Was a courier, set. 32, who began to
he affected with sciatica?" racking
sciatica"?two years previously, whilst
travelling on the Continent. He had
been cupped, leeched, and blistered?
used hot baths and cold baths, mineral
waters, &c. without the slightest benefit.
When abroad, he had undergone acu-
puncturation three times, with some
temporary benefit, but would not submit
to the introduction of the needles any
uiore, on account of the pain they occa-
sioned. For fourteen months prior to
his admission, his sufferings had been
very severe, and his health was a good
deal broken in consequence. He could
lie only in the horizontal position, and
referred the pain accurately to the course
of the great sciatic nerve. Mr. Earle
put him upon alterative doses of the
blue-pill, with some relief to the pain,
but not making progress under this plan,
acupuncturation was tried, but only with
slight effect. He was then ordered the
subcarbonateof iron, 9j. every four hours,
the dose being gradually raised to 3ss.
The medicine disagreed with his stomach,
and was discontinued. Mr. Earle now
applied a dozen moderate sized moxas
along the thigh, and with the happiest
effect, for the pain in the thigh never
returned. It was necessary subsequently
to re-apply the moxa on the loins in con-
sequence of some pain there, which was
not relieved by cupping and belladonna
plaster. On the 1st January, he was
made an out-patient, and continues so
well, that he talks of resuming his old
situation as courier.
We think the moxa is scarcely used
enough by surgeons in this country. In
some cases, it is decidedly preferable to
cupping, on account of the manipulation
and disturbance which the part must
necessarily undergo during the latter
process. In an inflamed joint, for in-
stance, we have again and again, seeu
cupping ordered, the surgeon not con-
sidering that the pressure of the glasses
and scarificator, and the disturbance done
to the joint, must inevitably-do, at least
as much mischief, as the local depletion
can do good. As far as we have seen,
however, the moxa is not so applicable
to the acute stages of inflammation, as
to the more chronic forms of sciatica?
old sprains?stiff joints after rheumatism,
and cases of that description.
7. GUY'S HOSPITAL.
Hydrocele treated on Baron
Larrey's Plan.*
The patient was a robust blacksmith,
set. .32, the subject of a large hydrocele,
which had appeared gradually after a
blow, inflicted twelve months previously.
* London Med. Gazette, No. 8.
' '^1
502 Periscope; or, Circumspective Review. [Feb. 9
The tumour was tapped by Mr. Key, on
the 8th December, and 22 ounces of fluid
drawn off. A common gum-elastic ca-
theter was then introduced through the
canula into the tunica vaginalis, secured
by adhesive plaster, and the canula with-
drawn. Severe pain in the testicle and
cord ensued, effusion of a gelatinous con-
sistence took place, and, on the 11th, the
instrument was removed, but a fresh se-
cretion of serum into the tunica vaginalis
being the consequence, the catheter was
again introduced on the 20th. On the
23d, decided symptoms of inflammation
had taken place, and it was again with-
drawn. Adhesive inflammation now came
on, and on the 21st January, the patient
was discharged, a trifling induration in
the tunica vaginalis only remaining.
As an experiment, this case is satisfac-
tory enough, but certainly the catheter
will never supersede the ordinary method
of injection for the cure of hydrocele.
The common operation is over in a few
minutes, is productive of but little pain,
and is exceedingly successful. This, on
the contrary, is tedious, painful, and the
result any thing but certain. There are,
however, some cases where injection has
failed, and where the surgeon is obliged
to resort either to incision, or the caustic
potash, both which operations are ex-
tremely severe, and not entirely devoid
of danger. It is in these cases, we think,
that Baron Larrey's operation is deserv-
ing of a trial, as it is much more mild
than either of the above, and not, by any
means, so likely to induce constitutional
disturbance.
Mr. Key has, also, treated a case of
" house-maid's knee" in the same man-
ner, and with the same success. The
trocar and canula were pushed into the
most depending part of the tumour, three
ounces of fluid, having a reddish-yellow
colour and partly coagulating by heat,
drawn off, the gum-elastic catheter intro-
duced into the cyst, and a roller applied
around the joint, which was kept cool by
evaporating lotions. Much inflammation
was induced, and, on the 12th day, the
catheter was removed, when pressure and
the emp. ammon. c. hydrarg. completed
the cure.
The treatment is only applicable to
bursse which contain fluid. In such cases,
the evacuation of the fluid by a puncture,
and subsequent active blistering, will not
unfrequently effect a cure. A very short
time ago, a woman was admitted into
St. George's Hospital, with a swelling,
the size of a pigeon's egg, in the right
ham. It had no pulsation, had an un-
dulatory fluctuating feel, and was not
very painful upon pressure. The patient
stated that it had been coming on for
eight years, had been once entirely re-
moved by mercurial ointment, but sub-
sequently returned, and was attended
with such pain as to completely lame
her. Mr. Brodie imagining it to be an
enlarged bursa, punctured it with a fine
needle, when there dribbled out about
an ounce of yellowish fluid, of an oily
consistence, which entirely coagulated
upon heat. A blister was applied, and
kept open by the savirie ointment, and
whenv we saw the patient last, the tumour
had almost entirely disappeared.
If, however, the bursal sac, instead of
containing fluid, has become converted,
as happens in a great many cases, into a
solid tumour, with hardly any cavity in
its centre, it is clear that neither the
introduction of a catheter, nor incision,
can be of any service. In such a case
the sac must be dissected out, and it is
really surprising how little disturbance
such an operation will cause in a tole-
rably healthy constitution. We lately
saw an instance where this operation was
performed by Mr. Rose at St. George's
Hospital, and the inflammation which
followed was exceedingly trifling- At the
same time, it cannot be denied that bad,
and even fatal, consequences will occa-
sionally follow the excision of the bursa,
and that, sometimes, without the sur-
geon being able to detect the why or
wherefore. Not very long ago we re-
corded a case of this kind, which hap-
pened to M. Velpeau, at the Hopital de
Perfectionnement.
8. ST. GEORGE'S HOSPITAL.
Pecueiau Affection of the Thigh.
Two cases of a peculiar affection of the
thigh, dependent on rheumatic inflam-
mation, and terminating in ulceration of
the cartilages, are related in the 8th No.
of the Medical Gazette. The symptoms
presented by both patients bear a very
close resemblance, and the affection ap-
^828] Furious Delirium. 503
pears to be an exceedingly severe, and
even dangerous one.
In either case the swelling came on
suddenly, and was marked by some pe-
culiar characters. It extended from the
Upper part of the thigh affected, over the
knee, for some little distance down the
}eg?it was not circumscribed, but still
Jts principal seat was evidently in the
lower part of the thigh and knee, above
and below which it gradually passed away
?it was tense?elastic?exquisitely pain-
ful on the slightest pressure, and of a
glossy, marbled white, bearing a very
striking resemblance to the description
of phlegmatia dolens. The patient could
not support the least motion of the knee,
but the pain, though partly in the joint,
appeared to be principally in the textures
around it. These, with considerable ir-
ritability and feverishness, were the symp-
toms presented, upon their admission, by
the patients, both of whom had been much
exposed to damp and cold, the first being
a common prostitute?the second, a wash-
erwoman.
We shall not follow up the details of
the individual cases; suffice it to say that,
in the first, which was under the care of
Mr. Brodie, leeches frequently repeated,
With calomel and opium, so as to affect
the mouth, were employed, and with tem-
porary benefit. Symptoms, however, of
ulceration of the cartilages of the knee-
joint supervened, and were attended with
the most intense suffering. Leeches,
blisters, fomentations and plasters of bel-
ladonna, and, lastly, a caustic issue were
employed, but with no permanent good
effect. Ulcers formed over the sacrum,
which somewhat relieved the pain in the
knee, but the left shoulder now became
affected with excruciating pain, and a bi-
lious diarrhoea came on, and carried off
the unfortunate patient. On dissection,
the cartilages of the knee-joint were found
extensively ulcerated, and the periosteum
of the femur, though not perceptibly
thickened, was easily separable from the
bone, which was preternaturally vascular.
The only remedy which had even a
temporary effect in this case, was the ca-
lomel and opium, and Mr. Brodie, in his
clinical lecture, stated that he had met
with symptoms precisely similar, in a page
in a nobleman's family, which were suc-
cessfully treated by the above remedies.
The case which we noticed in our first
fasciculus, (page 450) appears to us to
have been the same, or nearly the same,
affection, save that, there, the inflamma-
tion had not attacked the joint, and had
taken on the chronic, instead of the acute
form. The second case we do not deem
it necessary to relate at length here. It
is still under treatment by Mr. Keate,
and the reporter states, that the woman
" bids fair, from the similarity of symp-
toms, of constitution, and even of personal
appearance, to fall into the same state as
the other unfortunate patient did before
her."
9. ST. THOMAS'S HOSPITAL.
Furious Delirium Treated by
Mercury.
A young man, lately from the country,
but, for the last six weeks, in Barclay's
brevvhouse, was received into St. Tho-
mas's Hospital (having been three days
ill) with furious delirium, constant jac-
titation, spitting of viscid saliva, flushed
face, hot dry skin?answers questions in
a loud, hurried, and anxious manner?
pulse 60, full, and firm. Was bled, and
fainted when sixteen ounces were ab-
stracted?after which, he was more tran-
quil, but seemed much exhausted. Ten
grains of calomel every four hours. To-
wards evening, (5th Jan.) he became
more anxious, and ultimately furious.
Twenty-four leeches to the temples, with
another interval of quietude. At 5 o'olock
in the morning of the 6th, the delirium
again returned. Eighteen ounces of
blood were drawn, and produced syn-
cope, after which, he slept, and was then
more composed. But lie still spoke in a
hurried manner, and the pulse was 150,
easily compressible. The calomel in the
above-mentioned doses, was still taken
regularly. Had three dark, offensive,
and liquid motions. Evening of the 6th.
Is more furious and restless?flushed
face?constant spitting?pulse quick, not
firm. Leeches to the temples?blister
to the head. The calomel every three
hours. The mouth became sore at 8
o'clock in the morning of the 7th, and
" directly the gums were decidedly affect-
ed?he became tranquil." The pulse
fell to 80, and the patient rapidly re-
covered.?Medical Gazette.
504 Periscope; or, Circumspective Review. [Feb. 0
Remarks. We have no fault to find
with the practice here pursued by Dr.
Elliotson; but we have some doubts as
to the disease being pure phrenitis. We
have seen several instances of this kind,
where furious delirium was produced by-
intestinal irritation, and we think the
above case was one where the said irri-
tation bore a considerable share in the
production of the symptoms. Probably
too, the young man had been making free
with " Barclay's Entire," and a dash
of delirium tremens might have entered
into the composition of the malady. The
pulse at 60?the black stools?the vary-
ing state ,of the cerebral affection?the
syncope from small detractions of blood,
and the previous history of the case,
incline us to this opinion, but we may
be wrong.
Medical Jurisprudence?Coroner.
On Saturday, the 2d February, Mr. Bar-
rett Marshall read a learned and eloquent
paper on Medical Jurisprudence, more
especially in relation to wounds .and to
Coroner's inquests. There was no pro-
fessional discussion, but a stormy debate
ensued, respecting the propriety, or ra-
ther the necessity, of the office of Coroner
being vested in a medical man. This
subject has lately been canvassed in one
of the weekly journals, without much be-
nefit, we fear, on account of the intem-
perate and passionate manner in which
the question has been approached. For
our own parts, we never wish to see the
medical character mixed up with the ad-
ministration of the laws of the land. The
only ?power which the physician or sur-
geon should aspire to, is knowledge.
It was maintained by some of the mem-
bers, that the duty of the Coroner re-
quired no legal knowledge. This we ap-
prehend to be a mistake. In the article
Coroner, in Rees' Cyclopaedia, it is sta-
ted that his " authority is judicial and
ministerial"?that "he is to commit those
to prison, who are found guilty, by his
inquest, of murder or other homicide
?" he must, also, inquire concerning
?their lands, goods, and chattels, which are
forfeited." " Another branch of his of-
fice is to inquire concerning shipwrecks
?and find out who is in possession of
the goods." In short, we conceive that
the Coroner's office is a judicial situation,
which is by no means adapted to the medi-
cal character. But we admit?it cannot be
denied, that, in almost all cases of Coro-
ner's inquests, the knowledge of the
medical man is required. This know-
ledge may surely be put in requisition,
without any necessity for the surgeon
being himself a Coroner. The whole of
the objections now urged against Coro-
ner's inquests might be easily removed,
by the appointment of a well-informed
surgeon to assist the Coroner, not only
by careful examination of the body, but
by interrogating the witnesses on such
points as fall within his department. It
should be, and we believe it is, in the
power of the Coroner, to summon, in
difficult cases, several medical men, in
order to have the benefit of their united
knowledge. A great outcry is made, as
to the necessity of minute medical know-
ledge in the Coroner; but we apprehend
that, before we urge the legislature to
appoint medical men to the Coroner's
office, we should first see that medical
men are themselves properly qualified.
Minute medico-legal knowledge is neces-
sary, and yet medical jurisprudence forms
no part of the system of medical educa-
tion (if system it can be called) in this
country! Let us lay the axe to the root
of the tree?01 rather of the evil. The
whole profession should unite in one ge-
neral petition to the legislature, for a
regulation of medical instruction. One
uniform system of medical education
should be instituted, and enforced by law
?and then let individuals1 practise such
branches afterwards as their genius suited,
or their circumstances and inclinations
rendered eligible.
We shall immediately proceed to the
subject of medical education generally,
and hope to shew, in a calm and argu-
mentative manner, the baneful effects of
the present system?or rather systems,
in this country. We shall also endeavour
to direct the profession to the only legi-
timate path of reform?a parliamentary
investigation.

				

